# Bacteriocins of *Listeria monocytogenes* and Their Potential as a Virulence Factor

**DOI:** 10.3390/toxins12020103

**Published:** 2020-02-05

**Authors:** Sangmi Lee

**Affiliations:** Department of Food and Nutrition, Chungbuk National University, Cheongju, Chungbuk 28644, Korea; sangmilee@chungbuk.ac.kr

**Keywords:** *Listeria monocytogenes*, virulence, microbiota, bacteriocin, listeriolysin S (LLS), Lmo2776, monocins

## Abstract

Intestinal microbiota exerts protective effects against the infection of various bacterial pathogens, including *Listeria monocytogenes*, a major foodborne pathogen whose infection can lead to a disease (listeriosis) with a high fatality rate. As a strategy to mitigate the action of the intestinal microbiota, pathogens often produce antimicrobial proteinaceous compounds such as bacteriocins. In this review, we summarize the information currently available for the well-characterized *L. monocytogenes* bacteriocin listeriolysin S, with the emphasis on its intriguing mode of action as a virulence factor, which promotes the infection of *L. monocytogenes* by changing the composition of the intestinal microbiota. We then discuss another intriguing *L. monocytogenes* bacteriocin Lmo2776 that specifically inhibits the inflammogenic species, *Prevotella copri*, in the intestinal microbiota, reducing superfluous inflammation while weakening virulence. In addition, we describe relatively less studied phage tail-like *Listeria* bacteriocins (monocins) and elaborate on the possibility that these monocins could be involved in enhancing pathogenicity. In spite of the burgeoning interest in the roles played by the intestinal microbiota against the *L. monocytogenes* infection, our understanding on the virulence factors affecting the intestinal microbiota is still lacking, calling for further studies on bacteriocins that could function as novel virulence factors.

## 1. Introduction

*Listeria monocytogenes* is the only species in genus *Listeria* that causes a disease (listeriosis) in humans via contaminated foods [1,2,3]. Healthy individuals infected by *L. monocytogenes* usually experience febrile gastroenteritis; however, high-risk groups such as newborns, the elderly, the immunocompromised and pregnant women can develop severe, potentially fatal symptoms including meningitis, sepsis, stillbirths and abortions [1,2,3,4]. Also, listeriosis is characterized by a relatively high fatality rate (approx. 16%) compared with other important foodborne pathogens [5]. Meanwhile, *L. monocytogenes* is widely distributed in the environment and can persist in food-processing facilities, impeding the control of this pathogen [2,6,7]. Because of these features, *L. monocytogenes* has been considered a major concern for public health and food safety. 

During the last decades, numerous studies have successfully unraveled key players involved in *L. monocytogenes* pathogenicity [8,9,10,11,12]. Many of those well-characterized virulence factors are located in *L. monocytogenes* pathogenicity island 1 (LIPI-1) that is conserved among all pathogenic *L. monocytogenes* strains [8,9,10,11,12]. However, the entire picture of *L. monocytogenes* pathogenicity has not been understood, as exemplified by the fact that only very recently it was shown that the intestinal microbiota functions as the first line of defense against *L. monocytogenes* in the host intestine [13]. In fact, a protective role of the intestinal microbiota against bacterial infection (termed colonization resistance) has been reported for other enteric pathogens [14,15,16,17]. As a strategy to counteract the colonization resistance, pathogens often produce bacteriocins that are antimicrobial peptides/proteins that inhibit the growth of the closely related species while the producing bacteria are protected via immunity mechanisms [18,19,20,21]. In this review, we summarize the information currently available for the intensely investigated *L. monocytogenes* bacteriocin, listeriolysin S (LLS), in various aspects with emphasis on its role in pathogenicity. Also, we describe Lmo2776, another well-characterized *L. monocytogenes* bacteriocin involved in bacterial pathogenicity, as well as high-molecular-weight bacteriocins produced by *L. monocytogenes* (monocins) that have been far less studied while discussing their possible roles in pathogenicity. 

## 2. LLS, Streptolysin S (SLS)-Like Peptide in *L. monocytogenes*

LLS is a bacteriocin that possesses post-translationally modified amino acids and belongs to a subgroup of the family of thiazole/oxazole-modified microcins (TOMMs), which have been identified in various Gram-positive pathogens including *Streptococcus pyogenes* (SLS), *Staphylococcus aureus* (staphylolysin S; STS), and *Clostridium botulinum* (botulysin S or clostridiolysin S; BTS) [22,23,24,25,26,27,28]. Among these related bacteriocins, LLS displays the highest similarity to STS in the organization of the gene cluster and amino acid sequence of the encoded proteins, whereas SLS more closely corresponds to BTS [22,23,24,25,29]. This relationship is reflected in the fact that the proteins in the LLS operon exhibit 28.2%–53.8% identity to the corresponding *sts* gene products but 18%–24.7% to the proteins in the SLS-associated gene (*sag*) operon (data not shown) [25,29]. 

Most studies on this TOMM subgroup have been conducted on SLS, a constitutively expressed bacteriocin that is a potent hemolytic factor and a eukaryotic toxin [27,30,31]. As expected from these activities, SLS is a major virulence factor of *S. pyogenes* that enhances the bacterial survival in the phagosome during the infection and plays an indispensable role in the infection of skin and soft tissues as well as systemic spread of this pathogen [23,30]. Surprisingly, even though SLS is classified into bacteriocins, antimicrobial activity has not been reported for SLS, which does not kill intact bacteria [22,28,32]. 

Meanwhile, in agreement with the high similarity to SLS, BTS also exhibits hemolytic activity when reconstituted in the in vitro system containing the post-translational modification proteins (SagBCD) encoded in the *sag* operon (*sagABCDEFGHI*) or when complementing the deletion mutant of the SLS structural peptide gene (*sagA*) [22,23,24]. Similar findings were reported for STA, albeit only when the C-terminus of the STA structural peptide was fused with the SagA leader peptide [22,23,24]. Although BTS and STA have not been investigated as pathogenicity factors, these findings suggest that they might play important roles in the bacterial pathogenicity as demonstrated for SLS. 

**Figure 1:**
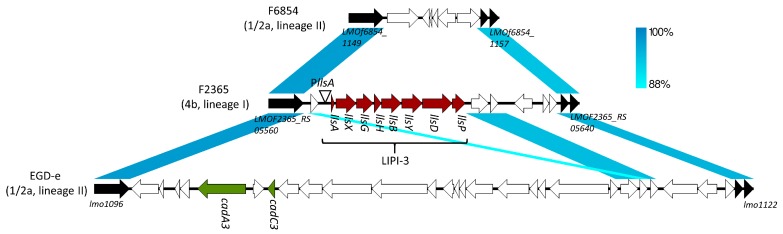
Comparison of LIPI-3 encoding the LLS biosynthetic cluster in the lineage I *L. monocytogenes* strain F2365 (accession no. NC_002973.6) and its corresponding region in the lineage II strains F6854 (accession no. AADQ01000003) and EGD-e (accession no. NC_003210.1). Homologous regions were identified via BLASTn and visualized with Easyfig in different shades of blue corresponding to the similarity of each homologous region [35,36]. The relationship between the blue shades and matching similarity values is shown in the gradient rectangle. Genes are shown as arrows. The *llsA* gene was not annotated in the F2365 whole-genome sequence available on National Center for Biotechnology Information; hence, its location was identified using the open reading frame identification function of SnapGene (GSL Biotech; available at snapgene.com), and BLASTp was employed to confirm that the translated sequence was indeed LlsA [35]. Genes conserved among all three strains are marked in black, while *lls* and cadmium resistance genes are signified in red and green, respectively. All the other genes are shown in white. The white triangle represents the promoter P*llsA* that was predicted by BPROM (http://www.softberry.com/berry.phtml?topic=bprom&group=programs&subgroup=gfindb) [37].

LLS was first identified via the homology with a post-translational modification gene (*sagB*) of the *sag* operon in a subset of lineage I isolates [22,25,30,31,33]. The LLS operon forms a genetic island, *L. monocytogenes* pathogenicity island 3 (LIPI-3), which is located in a genetic hot spot that harbors a conjugative integrative element containing cadmium resistance genes (*cadA3* and *cadC3*) in the lineage II strain EGD-e [25,34] (Figure 1). The LLS operon consists of eight genes: *llsA* encoding the structural peptide; *llsX* of unknown function; *llsGH* coding for an ATP-binding cassette transporter that could potentially export LLS; *llsBYD* encoding enzymes involved in post-translational modifications of the *llsA*-encoded peptide; and *llsP*, whose product is putatively a metalloprotease responsible for bacteriocin leader cleavage [22,25,29] (Figure 1). Although most of the *lls* genes shared homology with *sag* genes, *llsX* was specific to the genus *Listeria,* and its inactivation led to the loss of LLS activity, suggesting that LLS might possess features distinct from SLS, which will be discussed further in the subsequent sections [25,28,29]. 

## 3. LLS as a Virulence Factor

The genetic similarity of LLS to SLS, which is a major virulence factor of *S. pyogenes*, led to the intensive investigation of the effects of LLS on virulence using various animal models [23,25,30,38,39,40]. The initial evidence of the contribution of LLS to *L. monocytogenes* virulence was obtained via mouse intraperitoneal infection model. In this study, *llsB* deletion resulted in significant decrease in viable *L. monocytogenes* cells in the livers and spleens compared with the wild-type (WT) strain, suggesting that LLS is necessary for virulence of *L. monocytogenes* in vivo [25]. This finding was also supported with the mouse oral infection model showing that a LLS-present lineage I strain (F2365) was more virulent than the LLS-deficient lineage II strains (EGD-e and 10403S) and that *lls* genes (*llsA* and *llsB*) are required in the capability of *L. monocytogenes* to invade the intestine and to persist in the intestinal content [39]. 

Later, the role of LLS in *L. monocytogenes* deep organ colonization was specifically examined with the mouse intravenous infection model, in which the intestinal lumen was not involved in infection and only inner organs were infected [40]. In mice intravenously infected with the *llsA* deletion strain, bacterial loads did not change in spleens or livers compared with the F2365 WT strain, suggesting that the role of LLS is limited to the intestine and is not essential for *L. monocytogenes* infection once this pathogen crosses the intestinal barrier [40]. 

The findings obtained from the murine models, i.e., the role of LLS in the infection of *L. monocytogenes* within the intestine but not within the deep organs, was confirmed with an additional animal model (chicken embryos) that completely bypassed the intestinal infection route [38]. When this model was employed, no difference in virulence was observed between the LLS-present strain (F2365) and LLS-absent strain (EGD-e) [38]. Also, the *llsA* deletion and LLS constitutive expression strains displayed comparable virulence to the F2365 WT strain [38]. Taken together, LLS contributes to virulence only when the intestine is involved, and this phenomenon is closely related to the intrinsic characteristics of LLS to be further discussed in subsequent sections. 

Contribution of LLS to pathogenicity might account for its distinct distribution in the *L. monocytogenes* population. As mentioned previously, the island containing the LLS operon (LIPI-3) was detected exclusively by some strains of lineage I, a population group which is over-represented among human clinical isolates and includes serotype 4b responsible for many epidemic listeriosis outbreaks [25,33,41,42,43,44,45]. In fact, several serotype 4b outbreak strains harbor LIPI-3 [25]. Also, LIPI-3 was over-represented among the clones with higher infectious potential in the multilocus sequence typing-based survey of the isolates collected from France between 2005 and 2013 [46]. On this note, LLS is an additional virulence factor produced by the isolates preferentially associated with epidemic listeriosis outbreaks and hypervirulence. 

Unexpectedly, the LLS cluster was detected partially or in its entirety in several nonpathogenic *L. innocua* strains [33]. The *llsA*-targeting PCR revealed that ~60% of *L. innocua* strains were positive for *llsA* [25,33]. Often, other *lls* genes were missing, but several strains harbored the full-length LIPI-3 [33], suggesting that LIPI-3, which might either be present in the common ancestor of *L. monocytogenes* and *L. innocua* or might have been recently acquired, has been subjected to reductive evolution similarly to LIPI-1 and the virulence gene *inlA* [33,47,48]. It is noteworthy that the constitutive expression of the *lls* operon in *L. innocua* exhibited the LLS phenotype, suggesting that the LLS operon is functional in *L. innocua* [33]. Based on these findings, although LLS expression has been observed only in the host intestine to be discussed in the subsequent section [39,40], LLS might be expressed outside the host (most likely only in certain environmental niches) and might play a role unrelated to infection, possibly in outcompeting bacteria in the same environmental niche. 

## 4. LLS, a Bacteriocin That Modulates the Intestinal Microbiota

Because of the unique features of SLS as a cytotoxic and hemolytic virulence factor that promotes bacterial survival in the phagosome, but without antimicrobial activity [22,23,27,28,30,31,32], it was hypothesized that LLS might contribute to virulence via similar modes of action. However, interestingly, LLS does not kill eukaryotic host cells, as demonstrated by Quereda et al., who showed that cytotoxicity was comparable among the WT, *llsA*-deletion and LLS constitutive expression strains when the human epithelial cells and mouse macrophages were infected [40]. 

Along the same lines, although SLS improves eukaryotic cell infection by favoring pathogen survival in the phagosome [30], LLS plays a role neither in eukaryotic cell infection nor in vacuolar escape [40]. No difference was observed in the intracellular cell counts in macrophages and epithelial cells infected by the WT, *llsA* deletion and LLS constitutive expression strains [40]. Also, in the experiments employing the cellular staining techniques, the LLS constitutive expression strain in the *hly* (encoding hemolysin listeriolysin O (LLO) [8,9,11]) deletion background remained as impaired in the vacuolar escape as the parental strain [40]. Put together, unlike SLS, LLS is neither cytotoxic to eukaryotic cells nor required for eukaryotic cell infection [40]. This can explain why LLS does not contribute to the infection of deep organs [38,40]. 

Meanwhile, similar to SLS, LLS exhibits hemolysis on blood agar plates, which has often been used as a convenient tool to assess LLS activity [25,29,40]. However, its hemolytic activity is much less efficient compared with the major *L. monocytogenes* hemolysin LLO in vitro [8,9,11,40]. In agreement with this feature, neither the *llsA* deletion nor constitutive LLS expression strains differed in phagocytic clearance in human blood from the WT [40]. Also, no significant changes in red blood cell counts were observed in mouse intravenous infection model among the WT, *llsA* deletion and complementation strains [40]. All these findings indicate that the hemolytic activity of LLS has little impact on the survival of *L. monocytogenes* in the blood after infection and, hence, the overall pathogenicity of *L. monocytogenes*.

Surprisingly, contribution of LLS to pathogenicity originates from its antimicrobial activity, which is commonly found among bacteriocins but not reported for SLS [28,32,39,40]. Production of LLS reduced the growth of LLS-deficient *L. monocytogenes* strains and other Gram-positive bacteria including *Lactococcus lactis* and *S. aureus* [39]. The direct evidence for the cytotoxicity of LLS on target bacteria was provided by observing through electron microscopy the disrupted cell walls and cell lysis of *L. lactis* after co-culturing with the LLS constitutive expression strain [40].

**Figure 2:**
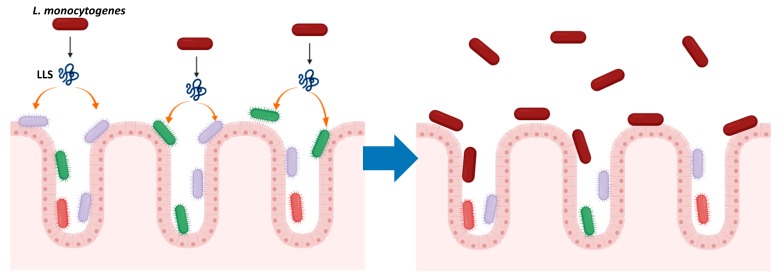
Mode of action of LLS as a virulence factor. LLS is not cytotoxic to eukaryotic cells nor increases cell infection; and its hemolytic activity minimally impacted *L. monocytogenes* infection in blood [40]. Instead, LLS promotes *L. monocytogenes* virulence via antimicrobial activity as a bacteriocin, i.e., by disrupting the intestine microbiota, thus enhancing *L. monocytogenes* colonization in the host intestine [39].

As a potent antimicrobial agent, LLS induces the changes in the host intestinal microbiota, thus providing the environment conductive to the augmented colonization of the intestine by *L. monocytogenes* [39] (Figure 2). When 16S ribosomal DNA sequencing was conducted on fecal samples from mice orally infected with the WT, *llsA* deletion and complementation strains, LLS significantly decreased the abundance of *Alloprevotella* and *Allobaculum* genera, which are deemed to protect the intestinal microbiota from *L. monocytogenes* [39]. Several *Alloprevotella* species produce acetic acid that impedes the growth of *L. monocytogenes,* whereas butyric acid produced by certain *Allobaculum* species reduces virulence factor production of *L. monocytogenes* at the transcriptional level [39,49,50,51,52]. This mode of action as a virulence factor via modulating the composition of intestinal microbiota accounts for the finding that the effect of LLS on pathogenicity is limited in the intestine; thus, it is not observed in animal infection models that circumvent the intestine [38,40]. 

Closely related to the importance of LLS as an intestine-specific virulence factor is that, while SLS is naturally expressed in routine laboratory conditions [30], LLS is not expressed outside the host [25]. Also, within the host, LLS expression is restricted within the intestine, not in the deep organs [39,40]. For instance, when in vivo expression of LLS was examined by orally infecting mice with the strain harboring the LLS operon promoter (P*llsA*) fused to the *lux* bioluminescence reporter system, the signal from the LLS promoter was found only in the intestine of the infected mice, not in other organs including liver and spleen [39]. In agreement with this finding, in mice intravenously infected with the same strain, bioluminescence was absent in deep organs (liver and spleen) but was only detected in the intestine of the mice as a result of the *L. monocytogenes* cells that were released back to the intestine from the gallbladder [40]. Attempts to identify the specific conditions where LLS is expressed have been made in several aspects, leading to confirmation that neither infection of eukaryotic cells in vitro nor intestinal microbiota, per se, induced LLS expression [39,40]. However, currently, the inducing signal for LLS expression in the host intestine remains undetermined and warrants further studies, which will provide insights into the regulation mechanisms of the LLS inside (and possibly outside) the host, thus facilitating in vitro research of LLS and devising strategies to reduce the pathogenicity of *L. monocytogenes* by turning off the expression of virulence genes. 

## 5. Lmo2776, Lactococcin 972-Like Peptide That Specifically Targets an Inflammation-Eliciting Bacterial Species in the Intestinal Microbiota, Leading to Impaired Virulence

**Figure 3:**
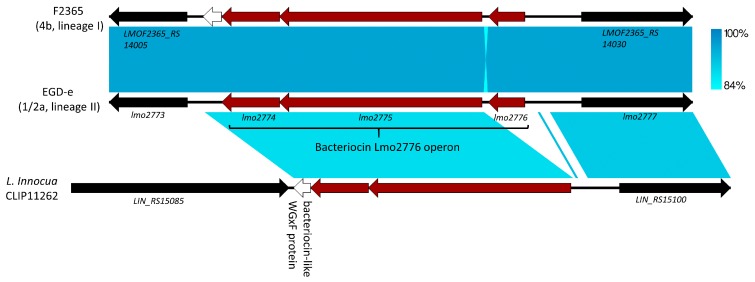
Comparison of the region containing the bacteriocin Lmo2776 synthesis operon in the lineage II strain EGD-e (accession no. NC_003210.1) and the corresponding region in the lineage I *L. monocytogenes* strain F2365 (accession no. NC_002973.6) and *L. innocua* CLIP11262 (accession no. NC_003212.1). This picture was drawn as detailed in Figure 1. Genes flanking the Lmo2776 synthesis operon are marked in black, and the bacteriocin-like WGxF protein gene found in the syntenic regions is in white. Genes within the Lmo2776 synthesis operon [53] are designated in red.

Lmo2776 is a bacteriocin recently identified in the reannotated EGD-e genome via its homology (amino acid similarity of 38%–47%) with the lactococcin 972 produced by *L. lactis* and its related bacteriocins found in various Gram-positive pathogens, i.e., *S. iniae*, *S. pneumoniae* and *S. aureus* [53] (Figure 3). Unlike LLS, Lmo2776 is expected to be secreted outside the cell without extensive modifications; in fact, the operon to which *lmo2776* belongs to includes only two other genes, *lmo2774* and *lmo2775*, whose products are putatively involved in immunity and transport functions, respectively [53] (Figure 3). Also, this bacteriocin was expressed in the laboratory condition (pronouncedly, in stationary phase) and was not found in *L. innocua* [53] (Figure 3). In addition to select lineage II strains such as EGD-e, *lmo2776* was only found in *L. monocytogenes* lineage I strains [53] (Figure 3). 

Along with LLS, Lmo2776 is one of two known bacteriocins produced by *L. monocytogenes* that influence bacterial virulence by modulating the intestinal microbiota [39,53]. However, contrary to LLS, Lmo2776 limits the intestinal infection of *L. monocytogenes* rather than contributes to *L. monocytogenes* pathogenicity [39,53]. The evidence on the negative impact of Lmo2776 on *L. monocytogenes* virulence was provided via the mouse oral infection model, in which *lmo2776* deletion increased the bacterial loads both in the intestine and in deeper organs [53]. Notably, Lmo2776 did not exert influence on bacterial growth in vivo in the mouse intravenous infection model, in which the intestine is not involved in the infection process, or in the absence of the intestinal microbiota, i.e., when germ-free mice were utilized [53]. Altogether, these findings imply that Lmo2776 impairs *L. monocytogenes* virulence only in the intestine, more specifically, by modifying intestinal microbiota. 

Among the bacteria constituting the intestinal microbiota, Lmo2776 specifically inhibits *Prevotella copri* that causes mucus erosion and exacerbates inflammation and bacterial infection in the intestine [53]. *P. copri* was identified as the target of Lmo2776 through 16S ribosomal DNA sequencing conducted on the mouse and in vitro reconstituted human microbiota, which revealed that Lmo2776 significantly decreased *P. copri* in both the mouse and human intestinal microbiota [53]. The inhibitory effect of Lmo2776 on *P. copri* was more directly demonstrated by the in vitro finding that *P. copri* growth was diminished by the culture supernatant containing Lmo2776 and the synthetic Lmo2776 peptide [53]. Intriguingly, when the germ-free mice were sequentially colonized by *P. copri* and *L. monocytogenes*, Lmo2776 decreased the number of *P. copri* and hampered *L. monocytogenes* virulence [53], providing compelling evidence that the decrease in *P. copri* abundance caused by Lmo2776 alone is sufficient to lower bacterial virulence. Altogether, these findings indicate that *L. monocytogenes* utilizes bacteriocins to modulate the intestinal microbiota not only to circumvent colonization resistance but also to prevent excessive inflammation at the expense of reduced virulence, highlighting the complicated interactions between the host, the microbiota and the enteric pathogens. Besides *P. copri*, Lmo2776 also kills *Bacillus subtilis*, which is often found in soil as well as in the human intestine [53], raising a possibility that Lmo2776 might confer fitness advantages outside the host, possibly in the soil.

## 6. Monocins: Tailocins of *L. monocytogenes* and Potential Virulence Factors

Besides LLS, *L. monocytogenes* strains produce monocins, which are various high-molecular-weight bacteriocins similar to phage tails (tailocins) [54,55,56]. Tailocins are widely identified within bacteria (both Gram-positive and Gram-negative bacteria), among which *Pseudomonas aeruginosa* has been extensively studied as a model organism [56]. Genes involved in tailocin synthesis exhibit a strong synteny with the tail assembly region of phages and also include those involved in gene regulation and cell lysis [56]. Tailocins are intracellularly produced upon induction of the SOS response (for instance, after treatment with mitomycin C or after UV irradiation) and are released via cell lysis, owing to the cell lysis cassette in the tailocin biosynthetic region to inactivate neighboring bacteria while not affecting the producing cells [54,55]. They are such potent bactericidal agents that only a single tailocin particle is sufficient to kill a bacterial cell [55]. Bacteria targeted by tailocins often belong to the same species of the producing cells; however, some tailocins show a broader bactericidal spectrum, influencing other species or genera [55]. Based on morphology, tailocins are divided into the R type, which shows morphological resemblance to the contractile tails of *Myoviridae* phages, and the F type, which is related to the non-contractile tails of the *Siphoviridae* phages and has been much less studied than R-type tailocins [55,56]. 

Monocins in *Listeria* were first reported in the 1960s and were employed as a typing tool often in combination with phage typing [57,58,59,60,61,62]. Early studies on monocins showed that these bactericidal molecules are frequently produced by *L. monocytogenes* (up to 76%) [58,59], and this finding was supported by the analysis of several whole-genome sequences of *L. monocytogenes*, *L. innocua* and *L. welshimeri*, which revealed that all the strains examined harbor a single monocin region [63]. 

Detailed molecular characterization of monocins was first conducted by Zink et al. in 1995, who investigated seven monocins from different *Listeria* species [54]. In this study, the phage tail-like structures of monocins were confirmed via electron microscopy, and the holin-lysin cassette in a *L. innocua* strain was associated with monocin production [54]. Also, DNA sequences of the monocin producers exhibited homology only with *Siphoviridae* phages, providing a line of evidence that monocins might belong to F-type tailocins [54]. 

**Figure 4:**
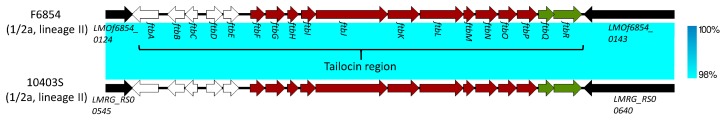
Monocin gene cluster *ftb* in F6854 (accession no. AADQ01000004) and 10403S (accession no. NC_017544). This picture was generated as described in Figure 1. Genes flanking the *ftb* cluster are in black. The regulatory, structural and lysis genes of the *ftb* cluster as designated by Lee et al. are marked in white, red and green, respectively [55].

Indeed, when the first monocin gene cluster, which is present in the serotype 1/2a strain F6854 and shares homology, albeit rather weak, with the *Siphoviridae* phage A118, was characterized in 2016, Lee and colleagues found that the monocin analyzed (M35152) was classified into a F-type tailocin; hence, the monocin gene cluster was designated *ftb* (for F-type bacteriocin) [55]. The *ftb* cluster consists of 18 genes including the *lmaA* gene, or antigen A gene (*ftbG*), encoding the monocin major tail protein that also derives immune response during infection and the most downstream holin-lysin cassette (encoded in *ftbQR*) involved in the lysis of the producing cells and extracellular release of monocin [55] (Figure 4). Lee et al. cloned and expressed the *ftb* cluster in *Bacillus subtilis* to confirm the lethality of M35152 and the role of the holin-lysin cassette in monocin release [55]. 

Of particular note is that the bactericidal range of a monocin can be modified via genetic engineering [55]. Fusing M35152 effective only against serotype 4b with the receptor-binding function of the A118 (infecting serotypes 1/2a, 1/2b and 1/2c) tail fiber resulted in a recombinant monocin with a wider killing range including serotypes infected by A118 [55]. The *ftb* cluster was also identified in the serotype 1/2a lab strain 10403S, and the role of the *ftb* holin-lysin cassette in cell lysis was confirmed using a variety of deletion constructs in 10403S [64] (Figure 4). 

In spite of the seemingly wider distribution than LLS and Lmo2776, our current knowledge on monocins lags behind these bacteriocins. To our knowledge, the role that monocins play in *L. monocytogenes* pathogenicity has not yet been elucidated; moreover, no tailocins have been identified that influence the virulence of enteric pathogens. However, it is sufficiently conceivable that monocins might be engaged in pathogenicity in similar manners to LLS and Lmo2776, that is, by changing the microbial landscape of the intestine so that *L. monocytogenes* can take a favorable position in establishing itself in the intestine amidst the competition with the intestinal microbiota or can avoid superfluous inflammation with the loss of virulence. In support for this speculation, the antigen A encoded in the above-mentioned *ftb* monocin region elicits the immune response, suggesting that monocins might be expressed during infection [65]. Moreover, genotoxic stress that could induce the SOS response has recently been reported to be present in the mouse intestine [66].

## 7. Conclusions

As a result of comprehensive research on *L. monocytogenes* pathogenesis, we have witnessed a considerable surge in our understanding on various virulence factors of *L. monocytogenes*, including the latest appreciation on the important role of the microbiota to defend against *L. monocytogenes* infection in the intestine. Hence, although bacteriocins have long been known in *L. monocytogenes*, current knowledge is also limited on their potential as a major means to impede the protective action of the intestinal microbiota, thereby increasing pathogenicity. In this regard, the research on LLS provides valuable insights into how a bacteriocin contributes to virulence, i.e., by modulating the intestinal microbiota under the strict, not yet known regulation that allows for expression only in the intestine of the infected host, while not being involved in deep organ infection. Also, the investigation of Lmo2776 revealed that bacteriocins can also be employed to evade excessive inflammation by specifically reducing the intestinal microbiota species promoting inflammation, although these actions might sacrifice virulence to some degree. Through similar mechanisms, some of phage tail-like monocins that are widely present in the *L. monocytogenes* population are likely to augment the colonization of this pathogen in the host or cut back inflammation at the cost of virulence. In spite of intriguing features unraveled by past and recent efforts, many questions still remain to be explored for all bacteriocins that are covered in this review (Table 1). Given the growing importance of the intestinal microbiota in the protection against *L. monocytogenes* infection and the scarcity of our current knowledge on the virulence factors affecting the intestinal microbiota, bacteriocins of *L. monocytogenes* are a research area worth pursing as a source of novel virulence factors and potential antimicrobials that could be utilized to control this important foodborne pathogen.

## Figures and Tables

**Table 1:** Bacteriocins in *L. monocytogenes.*

Name	Mechanism of Action	Gene Expression Condition	Targets	Effect on Virulence	Further Questions	References
LLS	ND ^1^	Host intestine	*Alloprevotella* and *Allobaculum*	+ ^2^	What signals induce LLS expression? What is the structure of the mature LLS? What is the mechanism of action as a bacteriocin? Is LLS expressed outside the host? What functions does LLS play in the environment?	[22,25,29,33,38,39,40]
Lmo2776	ND	Stationary phase in the laboratory condition	*P. copri* and *B. subtilis*	− ^3^	Why do *L. monocytogenes* strains harbor lmo2776? What signals induce the expression of Lmo2776? What is the structure of Lmo2776? What is the mechanism of action as a bacteriocin? What functions does it play in the environment?	[53]
Monocins including M35152	ND	SOS response-inducing conditions	*L. monocytogenes*	ND	Outside *L. monocytogenes*, what bacteria do monocins target? Do monocins contribute to the virulence of *L. monocytogenes*? Are monocins expressed inside the human intestine?	[54,55,57,58,59,60,61,62,63,64]

^1^ ND, not determined. ^2^ +, increase virulence. ^3^ −, decrease virulence.
